# Salt stimulates carbon fixation in the halophyte *Nitraria sibirica* to enhance growth

**DOI:** 10.48130/forres-0025-0004

**Published:** 2025-02-25

**Authors:** Lu Lu, Yuru Wang, Yuchang Chen, Liming Zhu, Xinru Wu, Jisen Shi, Jinhui Chen, Tielong Cheng

**Affiliations:** State Key Laboratory of Tree Genetics and Breeding, Co-Innovation Center for Sustainable Forestry in Southern China, Nanjing Forestry University, Nanjing 210037, China

**Keywords:** Salt adaptability, Halophyte, *Nitraria sibirica*, Carbon fixation, Photosynthesis

## Abstract

Soil salinity significantly inhibits plant productivity by adversely affecting photosynthesis and growth. *Nitraria sibirica*, a typical halophyte, exhibits strong salt tolerance. In this study, salt-treated *Nitraria sibirica* seedlings demonstrated more vigorous growth and a higher photosynthetic rate than untreated control seedlings. Transcriptome analysis revealed that the upregulated differentially expressed genes including *ribose 5-phosphate isomerase A*, *ribulose-bisphosphate carboxylase large chain,* and *malate dehydrogenase* in the leaves of *Nitraria sibirica* treated with 500 mM NaCl were significantly enriched in the 'Carbon fixation in photosynthetic organisms' pathway according to the Kyoto Encyclopedia of Genes and Genomes database. The promoters of these three photosynthetic differentially expressed genes were predicted to contain *cis*-regulatory elements responsive to light, abscisic acid, and ethylene. Notably, genes encoding 1-aminocyclopropane-1-carboxylate synthase, a key enzyme in ethylene biosynthesis, and ethylene-responsive transcription factors were significantly upregulated in *Nitraria sibirica* under 500 mM NaCl treatment. Furthermore, quantitative real-time PCR analysis confirmed that the expression of these differentially expressed genes was significantly upregulated in *Nitraria sibirica* leaves treated with 500 mM NaCl and 500 mM ethephon for 1 h. In contrast, the expression of these salt-induced differentially expressed genes was significantly downregulated in *Nitraria sibirica* leaves treated with 500 μM aminoethoxyvinylglycine, an ethylene biosynthesis inhibitor, in combination with 500 mM NaCl for 1 h. These findings suggest that the enhanced photosynthesis observed in *Nitraria sibirica* under salt stress is likely mediated by ethylene signaling, which regulates the expression of genes involved in carbon fixation, thereby promoting vigorous plant growth.

## Introduction

Soil salinization poses a significant threat to agricultural productivity and environmental sustainability by reducing water uptake, causing excessive accumulation of toxic ions, and generating reactive oxygen species (ROS), which collectively induce oxidative stress in plants. These detrimental effects adversely impact the growth of most plant species^[1,2]^. Although the negative impact of salinity on plant growth has been recognized for decades^[3]^, the issue is escalating due to ongoing global climate deterioration and the expansion of irrigation practices, leading to increasing areas afflicted by salinity. These challenges not only hinder plant development but also exacerbate broader ecological issues. Salt-affected soils markedly decrease the yield of conventional crops, raising serious economic concerns regarding global food shortage, especially as the world population is projected to reach 9.3 billion by 2050^[4−6]^. In response, extensive efforts have been undertaken to enhance the salt tolerance of plant species, thereby enabling more effective utilization of saline soils. Despite these endeavors, more plant species, known as glycophytes, remain highly sensitive to salinity^[3]^. Glycophytes can tolerate only low salt concentrations and have been predominantly used in studies exploring the mechanisms of salt stress responses, including model species such as *Arabidopsis thaliana*, tobacco, and rice^[7−11]^. In contrast, salt-tolerant plants, which are more appropriate models for studying salt adaptation, have been comparatively less investigated.

Photosynthesis is indispensable for plant growth and development, sustaining life on Earth. However, it is highly sensitive to abiotic stresses, including salinity, which limits the photosynthetic efficiency of plants^[12−14]^. As the foundation of plant biomass production, photosynthetic capacity declines under salt stress, directly reducing crop yield, and further threatening global food security^[14−16]^. Numerous studies have focused on photosynthesis-related genes to improve photosynthetic efficiency, enhance productivity, and increase ecological adaptability. Ribulose-1,5-bisphosphate carboxylase (RuBisCO), a critical enzyme for carbon fixation in photosynthesis, plays a key role in plant productivity by incorporating atmospheric carbon dioxide into biomass^[17−20]^. For example, research on the RuBisCO gene demonstrated that high expression of the gene encoding ribulose bisphosphate carboxylase large chain (rbcL) (RuBisCO large subunit) was induced by optimized iron concentrations, resulting in increased biomass in *Chlorella vulgaris*^[21]^. Under abiotic stresses, some species from arid regions utilize specific carbon fixation pathways such as the C4 pathway or crassulacean acid metabolism (CAM), to enhance photosynthesis and improve stress tolerance^[22,23]^.

Emerging evidence suggests that ethylene signaling plays a critical role in improving photosynthesis under abiotic stress conditions. For instance, ethephon application upregulated the *psbA* and *psbB* genes of photosystem II (PS II) in heat-stressed rice, enhancing photosynthesis and indicating that ethylene regulates photosynthesis through carbohydrate metabolism^[24]^. Similarly, transcriptome analysis of *Zoysia japonica* under cold stress revealed that ethephon-regulated genes are involved in chlorophyll metabolism, promoting chlorophyll content and improving plant growth^[25]^. Ethylene has also been implicated in mitigating the effects of salinity stress on photosynthesis. For example, ethylene alleviated glucose-mediated repression of photosynthesis in salt-stressed wheat^[26]^. In *Pusa vijay*, ethephon treatment enhanced growth, photosynthesis, and the activity of 1-aminocyclopropane carboxylic acid synthase (ACS), a key enzyme in ethylene biosynthesis. Notably, ethephon significantly improved seed germination and reduced hydrogen peroxide (H_2_O_2_) content under salt stress. Ethylene-responsive transcription factors (ERFs), known to regulate photosynthesis and plant development, also play essential roles in this process^[27]^. Collectively, these findings suggest that the ethylene is crucial for alleviating the inhibitory effects of abiotic stress on photosynthesis and promoting plant growth^[28]^.

*Nitraria sibirica* Pall. (*N. sibirica*), a highly salt-tolerant halophyte, exhibits remarkable adaptability to extreme drought and saline-alkali conditions. It is commonly found in sandy deserts, arid lands, and saline grasslands and is widely cultivated for stabilizing sand deposits and desalinizing saline soil^[29−33]^. Plants of the *Nitraria* genus provide medical value and economic benefits through their fruits and leaves. Importantly, *Nitraria* plants are considered ideal models for investigating the mechanisms of salt tolerance due to their unique physiological traits and genetic resources. The identification and expression of salinity-responsive genes from *N. sibirica* have been shown to enhance salt tolerance when expressed in transgenic plants^[34]^.

High salinity often represses plant growth by reducing photosynthetic rates. However, *N. sibirica* not only adapts to saline-alkali soils but also exhibits enhanced growth under certain high salt concentrations^[35,36]^. In this study, young seedlings of *N. sibirica* under salt stress displayed higher photosynthetic rates compared to control seedlings grown without salt. Transcriptome analysis of *N. sibirica* under salt conditions revealed significant upregulation of genes involved in carbon fixation and ethylene signaling pathways. These findings were further validated through treatments with ethephon and its biosynthesis inhibitor aminoethoxyvinylglycine (AVG), providing insights into the molecular mechanism underlying salt-induced photosynthesis in *N. sibirica*.

## Materials and methods

### Plant materials and treatments

Seeds of *N. sibirica* were generously provided by the Experimental Center for Desert Forestry of the Chinese Academy of Forestry (Bayannur 015200, Inner Mongolia, China). Before germination, the seeds were mixed with moist sand and subjected to cold stratification at 4 °C for two months. Following stratification, the seeds were removed from 4 °C storage and sown in pots containing a 1:1 mixture of soil and sand. The pots were placed in a growth chamber maintained at 22 °C with a 16-h light/8-h dark photoperiod to facilitate germination. To assess plant growth under salt stress, seedlings were irrigated with a 100 mM NaCl solution prepared using tap water immediately after germination. A separate set of seedlings was irrigated with tap water to serve as the controls. After two months, phenotypic data, including leaf size, plant height, and leaf number, were collected to evaluate the impact of the 100 mM NaCl treatment on *N. sibirica* growth. Each treatment was replicated three times, with each replicate comprising at least 15 seedlings.

### Determination of chlorophyll fluorescence parameters

To evaluate the effects of salt stress on photosynthesis, changes in P700 absorption at the Photosystem I (PS I) reaction center and chlorophyll fluorescence were simultaneously measured in control and 100 mM NaCl-treated seedlings using a portable Dual-PAM-100 Chlorophyll Fluorometer (Waltz, Germany)^[37]^. Before measurement, seedlings were dark-adapted in a dark room for 20−60 min. The third fully expanded leaf from the top was selected from each plant grown under normal or salt stress conditions for photosynthetic parameter analysis. Photosynthetic measurements were performed with three biological replicates, each consisting of 3−6 seedlings.

### RNA-seq

To identify genes responsive to salt stress, one-month-old *N. sibirica* seedlings were treated with 500 mM NaCl for 1 h, as the phenotypic effects of 100 mM NaCl require a longer duration to manifest. Leaves from salt-treated and control (untreated) plants were harvested for RNA sequencing (RNA-seq). Three biological replicates, each consisting of a single seedling, were used per treatment. Total RNA was extracted using the Eastep® Super Total RNA Purification Kit (Promega, Shanghai, China). RNA quantity and integrity were assessed using the RNA Nano 6000 Assay Kit of the Bioanalyzer 2100 system (Agilent Technologies, CA, USA). RNA-seq was performed on an Illumina HiSeq platform, generating 150-bp paired-end reads.

### Bioinformatic analysis

Clean reads, obtained by removing reads containing adapters, poly-N sequences, or low-quality bases, were used for downstream analyses. Trinity software (v2.6.6) was employed to assemble the clean reads into a reference transcriptome^[38]^. Differentially expressed genes (DEGs) were identified by comparing 500 mM NaCl-treated plants with control plants using the DESeq2 R package (1.20.0)^[39]^. DEGs were selected based on |Log_2_Fold change| > 0.5 and an adjusted *p*-value (*q*-value) < 0.05. The fragments per kilobase of transcript per million mapped reads (FPKM) values^[40]^ for each gene were calculated and used to construct expression heatmaps. Gene Ontology (GO) and Kyoto Encyclopedia of Genes and Genomes (KEGG) enrichment analyses of DEGs were performed using the GOseq R package (v1.10.0), and KOBAS software (v2.0.12), respectively, based on the hypergeometric distribution principle. Significance thresholds for GO and KEGG enrichment were set at *p* < 0.05. For promoter sequence *cis*-element analysis, 3,000 bp upstream of the start codon of target genes were extracted from the *N. sibirica* genome using TBtools. *Cis*-elements were predicted using the PlantCARE tool (http://bioinformatics.psb.ugent.be/webtools/plantcare/html/)^[41]^.

### Determination of relative gene expression by quantitative real-time PCR (qPCR)

To validate the expression of genes involved in photosynthesis and ethylene signaling, one-month-old *N. sibirica* seedlings were treated with 500 mM NaCl for 1 h. Additionally, seedlings were treated with 500 mM ethephon, or 500 μM AVG in combination with 500 mM NaCl for 1 h^[42]^. Leaves from treated seedlings were harvested for gene expression analysis. Total RNA was extracted using the Eastep® Super Total RNA Purification Kit (Promega, Shanghai, China), and genomic DNA was digested with DNase I provided in the kit. RNA concentration and integrity were assessed via ultraviolet spectrophotometry and agarose gel electrophoresis, respectively. First-strand cDNA was synthesized using HiScript III 1^st^ Strand cDNA Synthesis Kit (+gDNA wiper) (Vazyme Biotech, Nanjing, China). Relative expression levels were measured by quantitative real-time PCR (qPCR) using AceQ qPCR SYBR Green Master Mix (Vazyme Biotech, Nanjing, China) on a LightCycler®480 qPCR detection system (Roche, Basel, Switzerland) following the manufacturer's instructions. Gene expression levels were normalized to the expression of the *N. sibirica* actin gene^[43]^. Three biological replicates, each consisting of a single seedling, were used for each treatment. All primers used for qPCR are listed in Supplementary Table S1.

### Statistics testing and threshold for significance

Student's *t*-test was used to identify statistical differences using GraphPad Prism v8 (GraphPad Software). Statistical significance was determined at a 5% confidence level (*p* < 0.05).

## Results

### Enhanced growth of *N. sibirica* under salt stress

**Figure 1:**
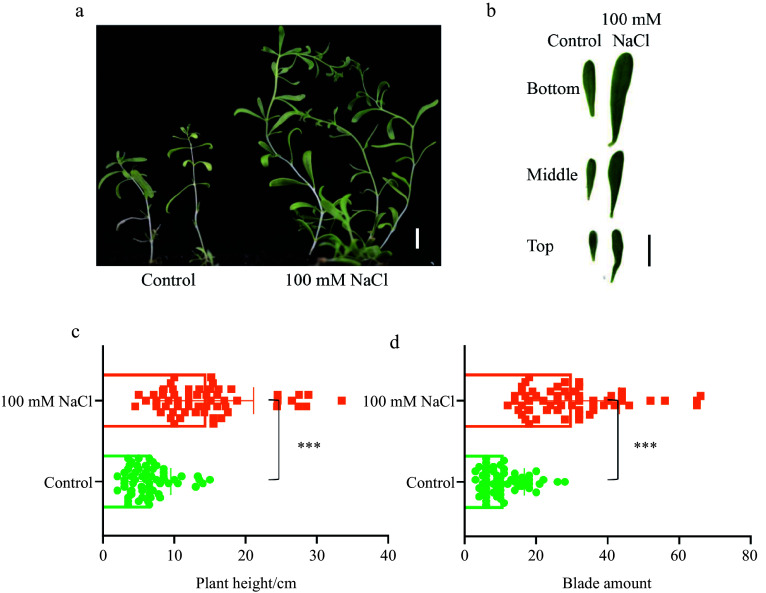
Growth performance of *Nitraria sibirica* under salt stress conditions. (a) Two-month-old *N. sibirica* seedlings grown in soil irrigated with tap water (Control) or 100 mM NaCl following germination. (b) Leaves collected from the basal, middle, and apical sections of seedlings as described in (a). Scale bar = 1 cm. (c) Plant height, and (d) leaf number of two-month-old seedlings grown under the conditions described in (a). Data are presented as means ± standard deviation (SD) from three biological replicates. Statistical significance was determined using a Student's *t*-test, where *** indicates *p* < 0.001.

Seedlings of *N. sibirica* grown under 100 mM NaCl conditions exhibited significantly more vigorous growth compared to control seedlings (Fig. 1a). Leaves from the basal, middle, and apical sections of salt-treated plants displayed larger surface areas than those from control plants (Fig. 1b). Additionally, salt-treated plants were notably taller and produced a greater number of leaves than their non-treated counterparts (Fig. 1c, d). These observations suggest that a salt concentration commonly employed in salt stress studies, validated as an effective treatment for most glycophytes, promotes the growth of the halophyte *N. sibirica*.

### Salt treatment enhances photosynthetic performance in *N. sibirica*

**Figure 2:**
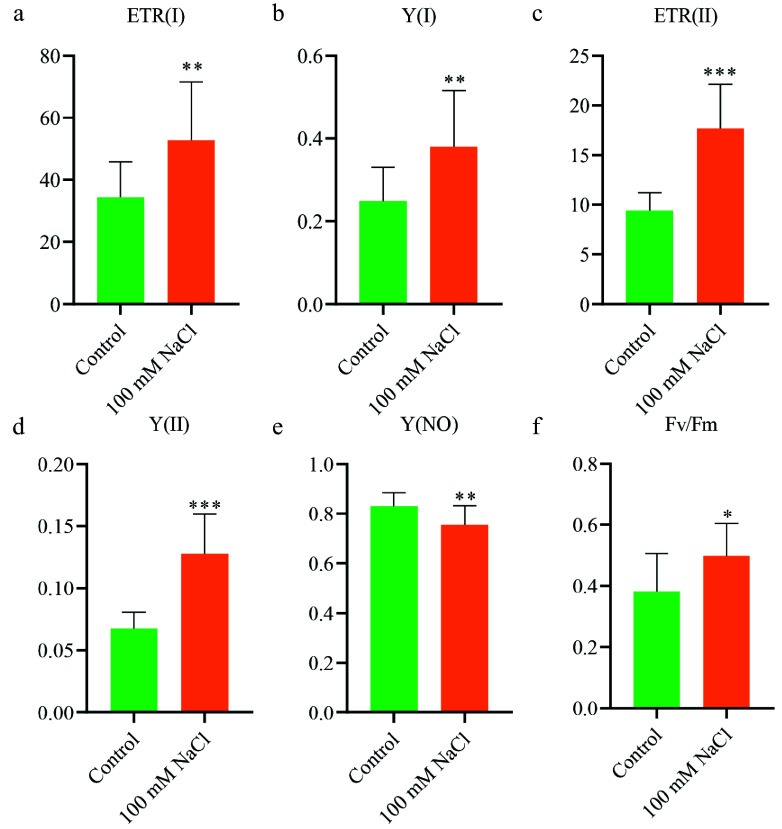
Enhanced photosynthetic activity of *N. sibirica* in response to salt treatment. (a) Electron transport rate of Photosystem I (ETR(I)); (b) Quantum yield of Photosystem I photochemistry (Y(I)); (c) Electron transport rate of Photosystem II (ETR(II)); (d) Quantum yield of Photosystem II photochemistry (Y(II)); (e) Quantum yield of non-photochemical quenching (Y(NO)); (f) Maximal quantum yield of PS II (F_*v*_/F_*m*_). Photosynthetic parameters were measured in seedlings grown under normal conditions (Control) or treated with 100 mM NaCl for two months. Data are presented as means ± standard deviation (SD) from three biological replicates. Statistical significance was determined using a t-test: * *p* < 0.05, ** *p* < 0.01, *** *p* < 0.001.

To evaluate the impact of salt treatment on photosynthesis, we measured the P700 redox state at the PS I reaction center and chlorophyll fluorescence *in vivo*^[44]^. Significant increases were observed in both the photosynthetic electron transport rate of Photosystem I (ETR(I)) and Photosystem II (ETR(II)) in salt-treated plants compared to controls (Fig. 2a, c). These results indicate enhanced electron transport activity in both PS I and PS II under salt stress.

Further analysis of quantum yields revealed that the quantum yield of PS I (Y(I)) and PS II (Y(II)) was significantly higher in salt-treated plants (Fig. 2b, d). Conversely, the quantum yield of non-photochemical quenching (Y(NO)) was reduced in salt-treated plants (Fig. 2e), indicating decreased energy dissipation. Notably, the maximal quantum yield of PS II (Fv/Fm) was significantly elevated in salt-treated plants (Fig. 2f), reflecting improved photochemical efficiency.

Collectively, these findings demonstrate that salt treatment enhances photosynthetic performance in *N. sibirica*, contributing to its robust growth under saline conditions.

### Salt-induced differential expression of genes in *N. sibirica*

**Figure 3:**
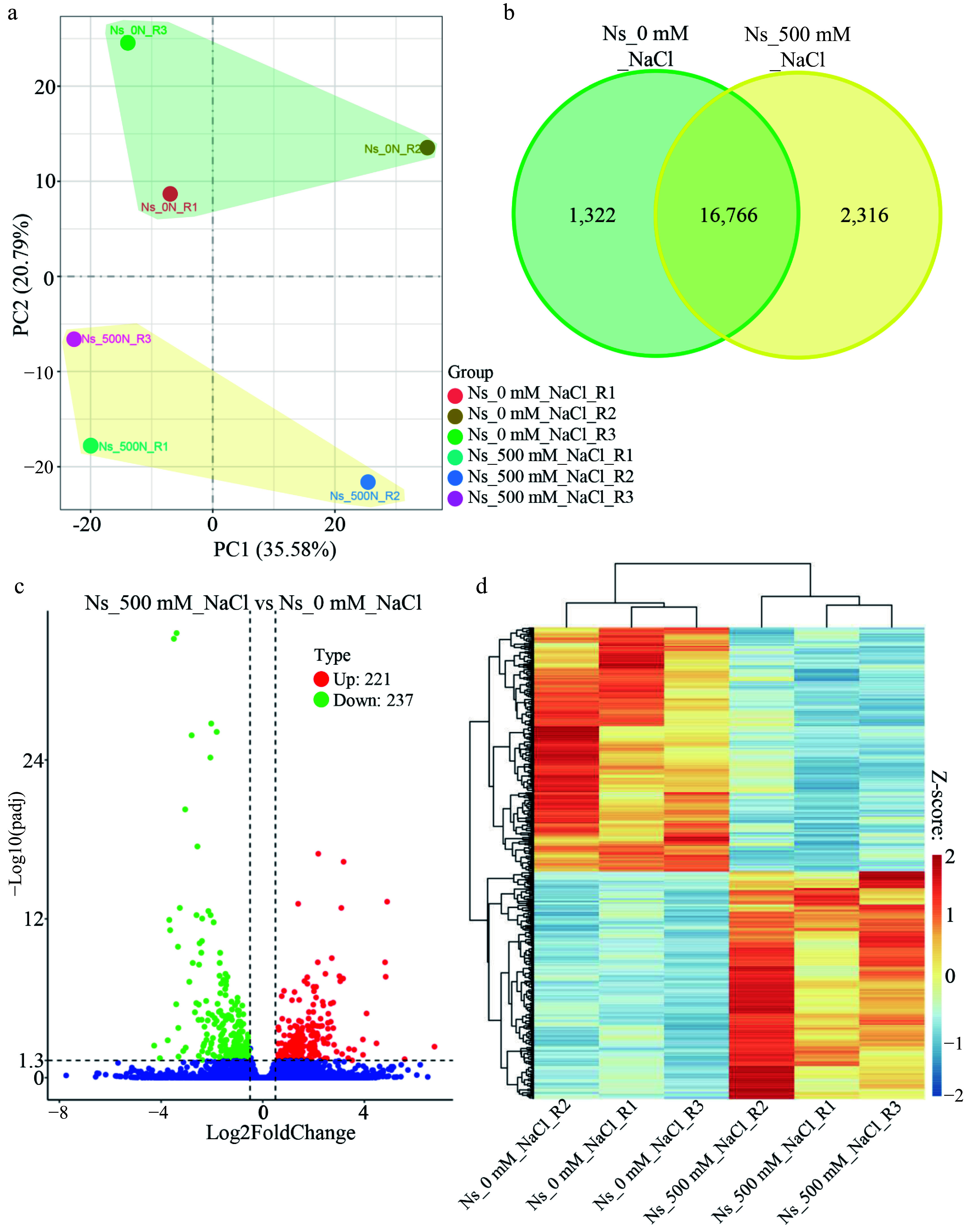
Salt induces differential gene expression in *N. sibirica*. (a) PCA plot of gene expression in leaves of control and salt-treated seedlings generated using the ggplot2 R package (Version 3.0.3). Seedlings were treated with 500 mM NaCl for 1 h compared to controls. (b) Venn diagram showing the number of genes uniquely expressed in control and salt-treated seedlings. Green shading represents control-specific genes, while yellow shading indicates salt-treated-specific genes. (c) Volcano plot displaying fold changes of differentially expressed genes (DEGs) between 500 mM NaCl-treated (Ns_500 mM_NaCl) and control (Ns_0 mM_NaCl) seedlings. Red and green dots represent upregulated and downregulated DEGs, respectively, based on |Log_2_Fold change| > 0.5, and an adjusted *p* value (*q* value) < 0.05. '221' and '237' denote the number of upregulated and downregulated DEGs. (d) Heatmap and hierarchical clustering of 458 DEGs induced by salt treatment. Expression levels are color-coded based on fragments per kilobase of transcript per million mapped reads (FPKM), scaled by row using Z-scores. Red and blue indicate high and low expression, respectively.

Given the enhanced growth phenotype of *N. sibirica* under 100 mM NaCl, we hypothesized that salt acts as a signaling molecule regulating the expression of growth-related genes. To determine whether photosynthesis-regulating genes respond rapidly to salt stress, we performed transcriptome analysis on seedlings treated with 500 mM NaCl for 1 h. Principal component analysis (PCA) performed using the ggplot2 R package (Version 3.0.3) revealed distinct clustering of biological replicates, with control and salt-treated samples from separated groups (Fig. 3a). Transcriptome sequencing identified 1,322 and 2,316 genes specifically expressed in control and salt-treated seedlings, respectively (Fig. 3b). Comparative analysis using the DESeq2 R package identified 458 DEGs, comprising 221 upregulated and 237 downregulated genes (Fig. 3c). Hierarchical clustering of these DEGs demonstrated clear separation between salt-treated and control samples (Fig. 3d), indicating that 1-h exposure to 500 mM NaCl induces significant substantial changes in gene expression in *N. sibirica*.

### DEGs regulate carbon fixation pathways in *N. sibirica*

GO enrichment analysis of the DEGs revealed significant enrichment in molecular functions related to transcription factor activity, hydrolase activity, and ubiquitin-protein transferase activity (Supplementary Fig. S1a). Separating the DEGs into upregulated and downregulated groups, we found that ubiquitin-protein transferase activity was enriched among downregulated DEGs, while transcription factor and hydrolase activities were enriched among upregulated DEGs (Supplementary Fig. S1b, c).

KEGG pathway analysis using the clusterProfiler tool indicated that DEGs were significantly enriched in pathways including 'Plant hormone signal transduction', 'Plant-pathogen interaction', 'Circadian rhythm - plant', and 'Carbon fixation in photosynthetic organisms', as well as various metabolic pathways (Supplementary Fig. S2a). Notably, the 'Carbon fixation in photosynthetic organisms' pathway was specifically enriched among upregulated DEGs (Supplementary Fig. S2b), correlating with the observed increase in photosynthetic rates under salt stress.

Further investigation identified the upregulation of genes involved in key carbon fixation pathways. Genes encoding ribose 5-phosphate isomerase A (rpiA) and ribulose-bisphosphate carboxylase large chain (rbcL), integral to the Calvin-Benson cycle, were upregulated in salt-treated plants (Supplementary Fig. S3). Additionally, the gene encoding malate dehydrogenase (maeB), involved in Crassulacean acid metabolism (CAM) and the C4-dicarboxylic acid cycle, was significantly upregulated in *N. sibirica* leaves under salt stress (Supplementary Fig. S3). These results suggest that salt stress enhances photosynthesis by activating gene expression related to carbon fixation pathways.

### ERFs potentially regulate carbon fixation genes

**Figure 4:**
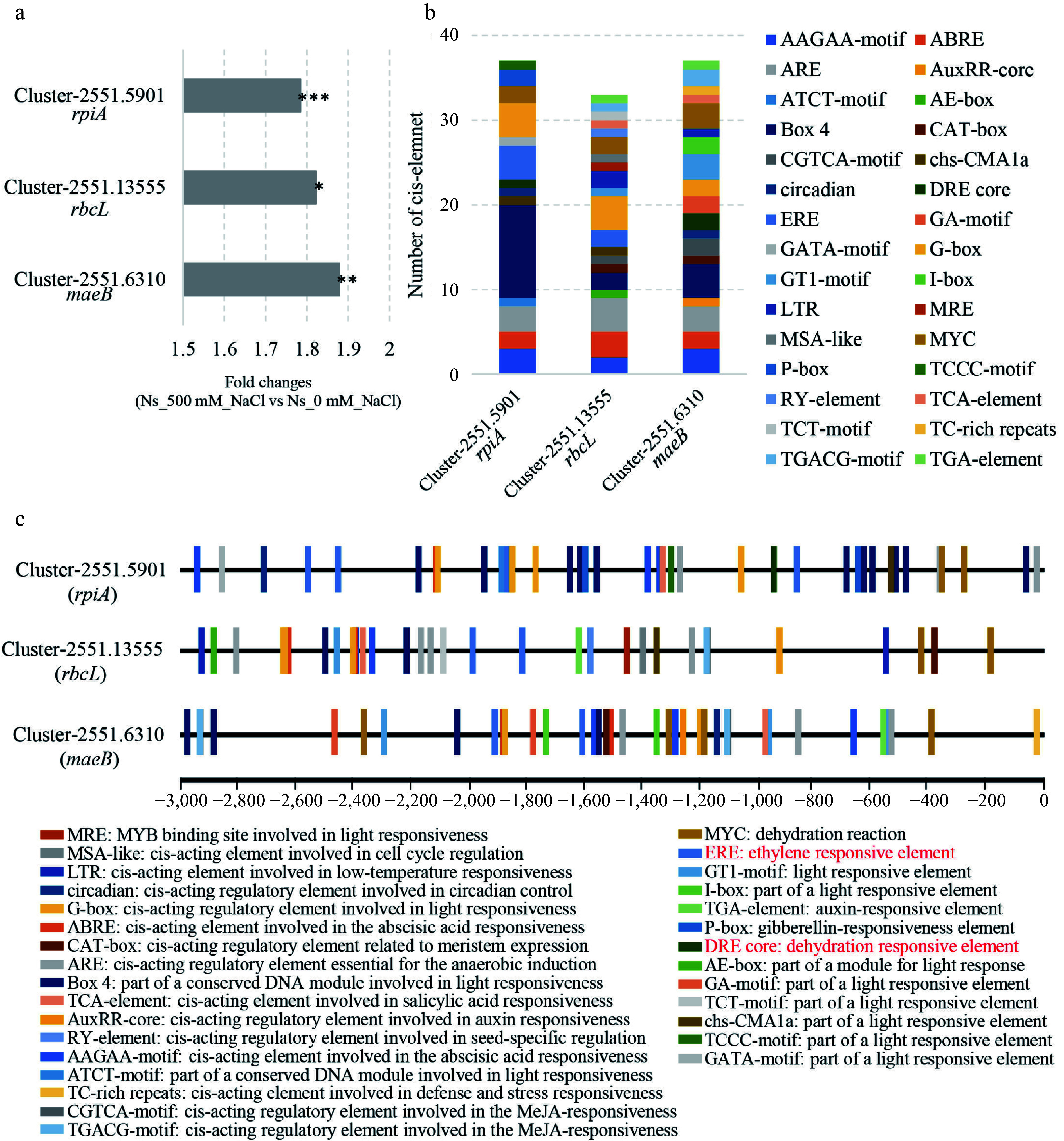
Identification of specific *cis*-elements in the promoter regions of DEGs involved in carbon fixation. (a) Fold changes in the expression of *rpiA*, *rbcL*, and *maeB* in *N. sibirica* treated with 500 mM NaCl (Ns_500 mM_NaCl) compared to control (Ns_0 mM_NaCl). (b) Number of *cis*-acting elements identified in the promoter regions of *rpiA*, *rbcL*, and *maeB*. (c) Distribution of *cis*-acting element in the 3 kb upstream regions of *rpiA*, *rbcL*, and *maeB*. Elements responsive to light, low temperature, abscisic acid, salicylic acid, auxin, methyl jasmonate (MeJA), gibberellin, and ethylene are indicated by different colored boxes.

Transcriptome data revealed that the expression levels of *rpiA*, *rbcL*, and *maeB* were increased by 1.78-, 1.82-, and 1.87-fold, respectively, in salt-treated plants compared to controls (Fig. 4a). To identify potential transcriptional regulators of these genes, we analyzed the *cis*-elements present within their promoter regions. A 3 kb sequence upstream of the start codon (including the 5' untranslated region) was extracted from the *N. sibirica* genome (Supplementary Table S2). *Cis*-element analysis identified various regulatory elements responsive to light, low temperature, abscisic acid, salicylic acid, auxin, methyl jasmonate (MeJA), gibberellin, and ethylene within the promoters of *rpiA*, *rbcL*, and *maeB* (Fig. 4b, c). Notably, these promoters share common *cis*-elements responsive to light, abscisic acid, and ethylene.

**Figure 5:**
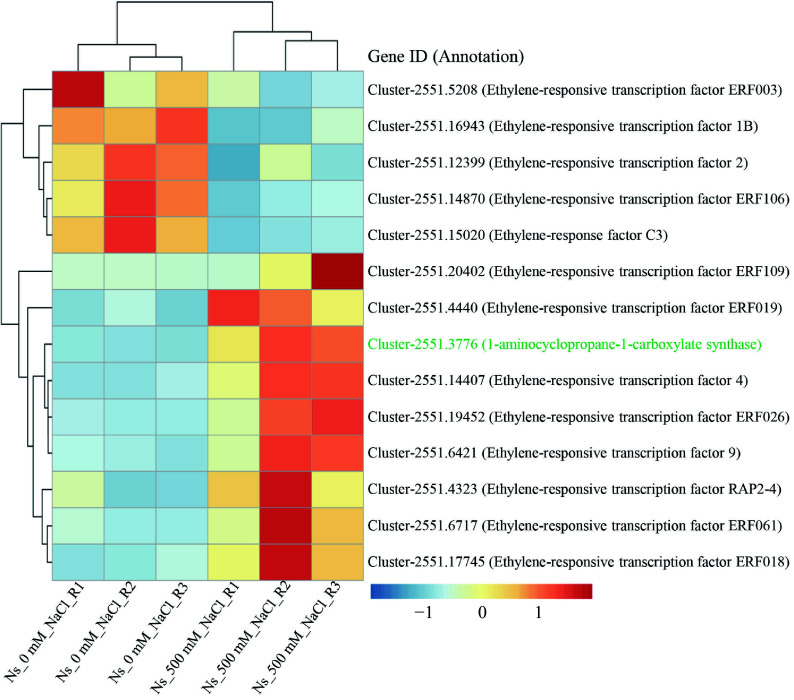
Salt treatment induces DEGs involved in ethylene signal transduction. Heatmap (right) and clustering analysis (left) of differentially expressed genes (DEGs) related to ethylene biosynthesis and response. Gene expression patterns are color-coded based on FPKM values scaled by row using Z-scores, ranging from low (blue) to high (red). 'Ns_0 mM_NaCl' refers to control seedlings, while 'Ns_500 mM_NaCl' refers to seedlings treated with 500 mM NaCl. Gene IDs and annotations are displayed alongside the heatmap. The 1-aminocyclopropane-1-carboxylate synthase (ACS), highlighted in green, catalyzes the rate-limiting step in ethylene biosynthesis.

Given the role of ERFs in regulating photosynthesis and plant development^[27]^, we examined the expression of ERF genes in our transcriptome data. Several ERF genes showed significant upregulation in salt-treated plants (Fig. 5). Furthermore, *cis*-elements known to be binding sites for ERF family transcription factors, such as the DRE core, ERE, and ATCTA-motifs, were present in the promoters of *rpiA*, *rbcL*, and *maeB* (Fig. 4b, c). Additionally, the gene encoding 1-aminocyclopropane-1-carboxylate synthase (ACS), which catalyzes the rate-limiting step in ethylene biosynthesis, was significantly upregulated in *N. sibirica* treated with 500 mM NaCl. Taken together, these results suggest that salt-induced upregulation of *rpiA*, *rbcL*, and *maeB* may be mediated by ethylene signaling through the activation of ERF transcription factors, thereby enhancing carbon fixation and photosynthesis to promote plant growth under salt stress conditions.

### Ethylene biosynthesis inhibition suppresses salt-induced gene expression

**Figure 6:**
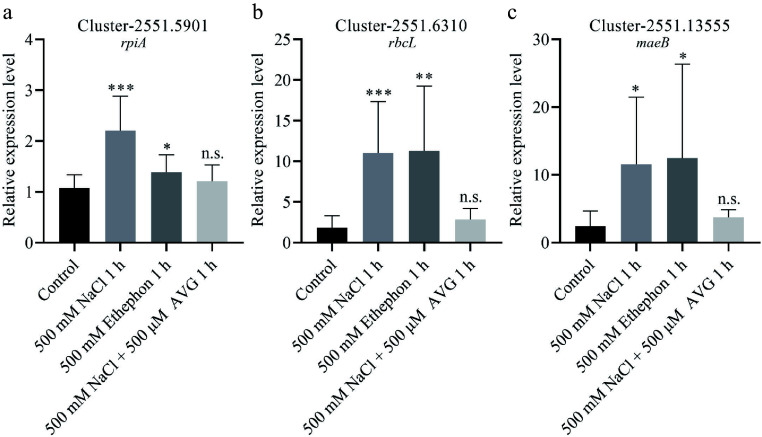
DEGs involved in carbon fixation positively responded to salt and ethephon treatment. (a) Relative expression levels of *rpiA*, (b) *rbcL*, and (c) *maeB* were analyzed in seedlings treated with 500 mM NaCl, 500 mM ethephon, and 500 μM aminoethoxyvinylglycine (AVG) in combination with 500 mM NaCl for 1 h. Seedlings treated with 0 mM NaCl served as the control (Control).

**Figure 7:**
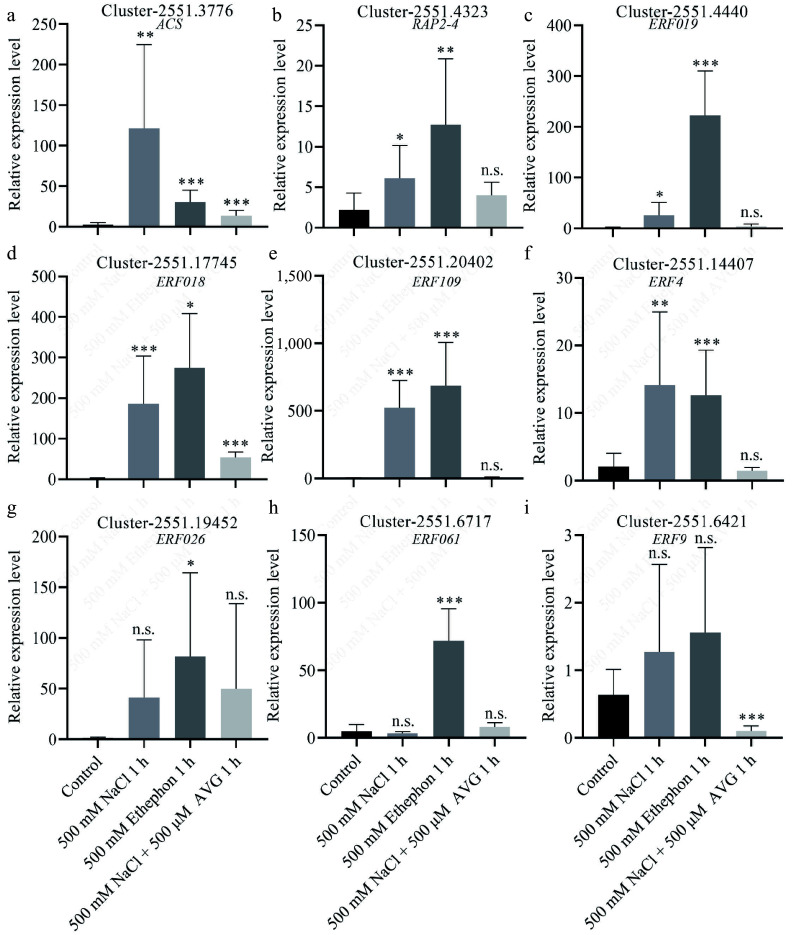
DEGs involved in ethylene signaling positively respond to salt and ethephon treatments. (a) Relative expression levels of genes encoding 1-aminocyclopropane carboxylic acid synthase (ACS), (b) Ethylene-responsive transcription factor (ERF) RAP2-4, (c) ERF109, (d) ERF018, (e) ERF109, (f) ERF4, (g) ERF026, (h) ERF061, and (i) ERF9. Expression was measured in seedlings treated with 500 mM NaCl, 500 mM ethephon, and 500 μM aminoethoxyvinylglycine (AVG) in combination with 500 mM NaCl for 1 h, respectively. Control seedlings were treated with 0 mM NaCl.

To validate the transcriptome findings, we performed qPCR on *N. sibirica* seedlings treated with 500 mM NaCl for 1 h. The expression of DEGs involved in carbon fixation, including *rpiA*, *rbcL*, and *maeB*, was significantly upregulated by 2.2-, 11.0-, and 11.6-fold, respectively, in salt-stressed plants compared to controls (Fig. 6a−c). Additionally, genes related to ethylene signaling, including Cluster-2551.3776 (encoding ACS) and eight ERF genes, were markedly upregulated in response to salt treatment (Fig. 7a−i). Consistent trends were observed in seedlings treated with 100 mM NaCl or 400 mM NaCl for 3 d (Supplementary Fig. S4)^[45]^.

To determine whether ethylene mediates the salt-induced expression of genes, we treated seedlings with ethephon (an ethylene-releasing compound) and AVG (an ethylene biosynthesis inhibitor) in combination with 500 mM NaCl. qPCR results revealed that photosynthesis-related DEGs were upregulated following ethephon treatment, mirroring the expression patterns observed under 500 mM NaCl treatment (Fig. 6a−c). In contrast, co-treatment with AVG and NaCl abolished the upregulation of these genes (Fig. 6a−c). Similarly, DEGs involved in ethylene signaling were induced by NaCl or ethephon alone but not when AVG was present alongside NaCl (Fig. 7). These results indicate that ethylene signaling is essential for salt-induced expression of genes involved in photosynthesis and ethylene response.

### Salt stress promotes starch accumulation in *N. sibirica* leaves

**Figure 8:**
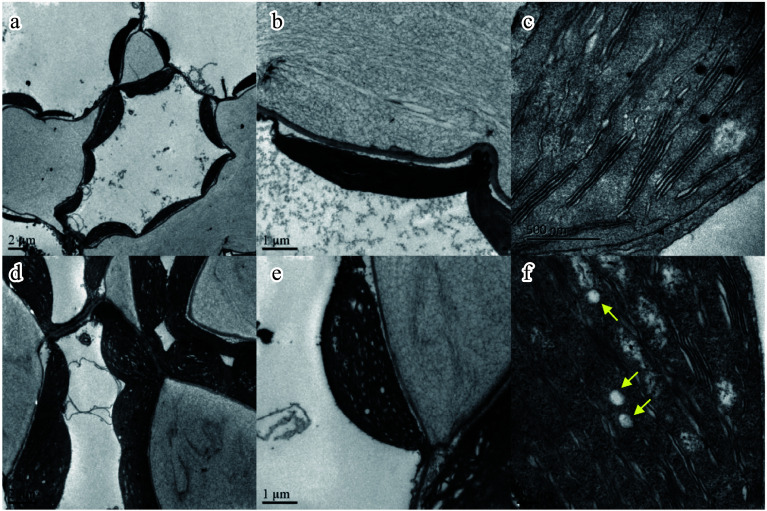
*N. sibirica* photosynthesis and chloroplast structures under salt treatment. (a) Transmission electron micrographs of mesophyll cells with chloroplasts under control conditions, and (d) after treatment with 500 mM NaCl for 1 h. (b) Detailed chloroplast structure under normal conditions, and (e) following 500 mM NaCl treatment. (c) Starch granules, indicated by yellow arrows, in chloroplasts of control plants, and (f) salt-treated plants.

Chloroplasts are crucial for starch synthesis, linking carbon fixation to chloroplast structure and function^[46,47]^. Transmission electron microscopy (TEM) was employed to examine chloroplast's morphology in *N. sibirica* leaves under normal and salt stress conditions. Chloroplasts in mesophyll cells maintained close interactions with the cell membrane in both control and salt-treated plants (Fig. 8a, d). Under salt stress, chloroplasts appeared swollen with larger surface areas; however, the fundamental chloroplast structure remained intact, and the outer membrane retained a regular shape after 500 mM NaCl for 1 h (Fig. 8b, e). Importantly, salt-treated plants accumulated more starch grains within their chloroplasts compared to control plants (Fig. 8c, f). These observations suggest that salt-induced enhancement of photosynthesis leads to increased starch biosynthesis and accumulation in *N. sibirica* leaves.

## Discussion

Tolerance to high salinity is a critical factor determining plant adaptation to saline environments. Most plant species experience reduced growth under saline conditions due to osmotic stress and the toxic accumulation of Na^+^ ions within their tissues^[48−50]^. However, certain highly salt-tolerant halophytes not only withstand high salinity without significant morphological changes but also thrive across a broad range of salt concentrations that typically inhibit the growth of glycophytes^[35,51,52]^. In our study, the halophyte *N. sibirica* exhibited remarkable salt adaptability, even at the seedling stage. Utilizing a 100 mM NaCl solution, we observed that two-week-old seedlings subjected to salt treatment displayed vigorous growth compared to those irrigated with tap water (Fig. 1). This observation is consistent with previous reports indicating that two-month-old seedlings treated with 100−200 mM NaCl for 14 d showed enhanced growth metrics, including increased plant height and leaf number, underscoring the robust salt tolerance of this species^[35]^.

Salinity typically imposes a combination of osmotic stress, ionic toxicity, and oxidative damage, which collectively lead to reduced photosynthetic rates, cellular damage, and metabolic imbalances, ultimately inhibiting plant growth and productivity^[53]^. Photosynthesis is a highly dynamic process that rapidly responds to environmental fluctuations^[54]^. Under abiotic stresses, declines in photosynthetic capacity are directly linked to reduced plant yield. Salinity impairs photosynthesis through both stomatal closure and biochemical limitations, disrupting light partitioning in PS II and increasing the dissipation of excess excitation energy via non-regulated mechanisms^[55]^.

In our study, transcriptomic analysis of salt-stressed *N. sibirica* upregulated genes involved in carbon fixation. Notably, the expression of the *rbcL* gene, encoding the large subunit of RuBisCO, was upregulated by 1.82-fold in salt-treated plants compared to controls (Fig. 4a). RuBisCO is a key enzyme in the Calvin-Benson cycle, pivotal for carbon assimilation during photosynthesis. The enhanced expression of *rbcL* correlated with the observed increases in photosynthetic rates and vigorous growth in salt-treated *N. sibirica*, suggesting an adaptive enhancement of the photosynthetic machinery under saline conditions.

Furthermore, salt treatment induced a 1.87-fold increase in the expression of *maeB*, which encodes malate dehydrogenase (oxaloacetate-decarboxylating) (NADP^+^) in *N. sibirica* (Fig. 4a)^[56]^. This enzyme is one of the three decarboxylases involved in carbon fixation pathways such as the C4 and CAM pathways, alongside malate dehydrogenase (MDH) and phosphoenolpyruvate carboxykinase (PEPC)^[57]^. Previous studies have demonstrated that many plant species possess the genetic capacity to employ both C3 and C4 or C3 and CAM photosynthetic pathways^[58]^. For instance, *Tamarix ramosissima* (*T. ramosissima*) utilizes C3 photosynthesis under normal conditions and shifts to CAM photosynthesis under extreme drought stress, with genes like *PEPC* and *MDH* being highly upregulated to facilitate this transition^[23]^. Moreover, overexpression of essential C4 photosynthesis genes, including *PEPC* and *MaeB,* in C3 model plants such as *Arabidopsis*, tobacco, and rice has been shown to enhance photosynthetic efficiency and confer increased tolerance to various abiotic stresses^[21,58]^. The upregulation of *maeB* in *N. sibirica* under salt stress may represent a similar adaptive mechanism, enabling the species to optimize carbon fixation and maintain metabolic balance in saline environments. This dual capacity for flexible carbon fixation pathways likely contributes to the exceptional salt tolerance and growth performance observed in *N. sibirica*.

## Conclusions

In this study, we conducted a comprehensive transcriptomic analysis of the halophyte *N. sibirica* under normal and salt stress conditions. Our findings reveal significant upregulation of ERFs in salt-treated *N. sibirica*. These ERFs are poised to regulate the expression of genes involved in carbon fixation, thereby facilitating the observed enhancement in photosynthetic efficiency and robust growth under saline stress. The identification of these salt-responsive genes associated with photosynthesis provides insights into the molecular mechanisms underpinning salt-induced growth promotion in halophytes. Additionally, this research highlights the ecological and economic potential of *N. sibirica* as a valuable halophyte species for cultivation in saline environments, offering avenues for agricultural practices in salt-affected regions.

## SUPPLEMENTARY DATA

Supplementary data to this article can be found online.

## Data Availability

All data generated or analyzed during this study are included in this published article and its supplementary information files. The raw RNA-seq data are publicly accessible through the NCBI database under BioProject accession number PRJNA904849.
