# Supplementary material for: Salt stimulates carbon fixation in the halophyte Nitraria sibirica to enhance growth
Source: For Res (Fayettev). 2025 Feb 25;5:e004. doi: 10.48130/forres-0025-0004 (PMC11922184; doi:10.48130/forres-0025-0004)
Supplement: Supplementary file 1 — Supplementary data to this article can be found online. [file forres-0025-0004-Supplementary.zip › 10.48130_forres-0025-0004-Suppl-FigureS2.pdf]

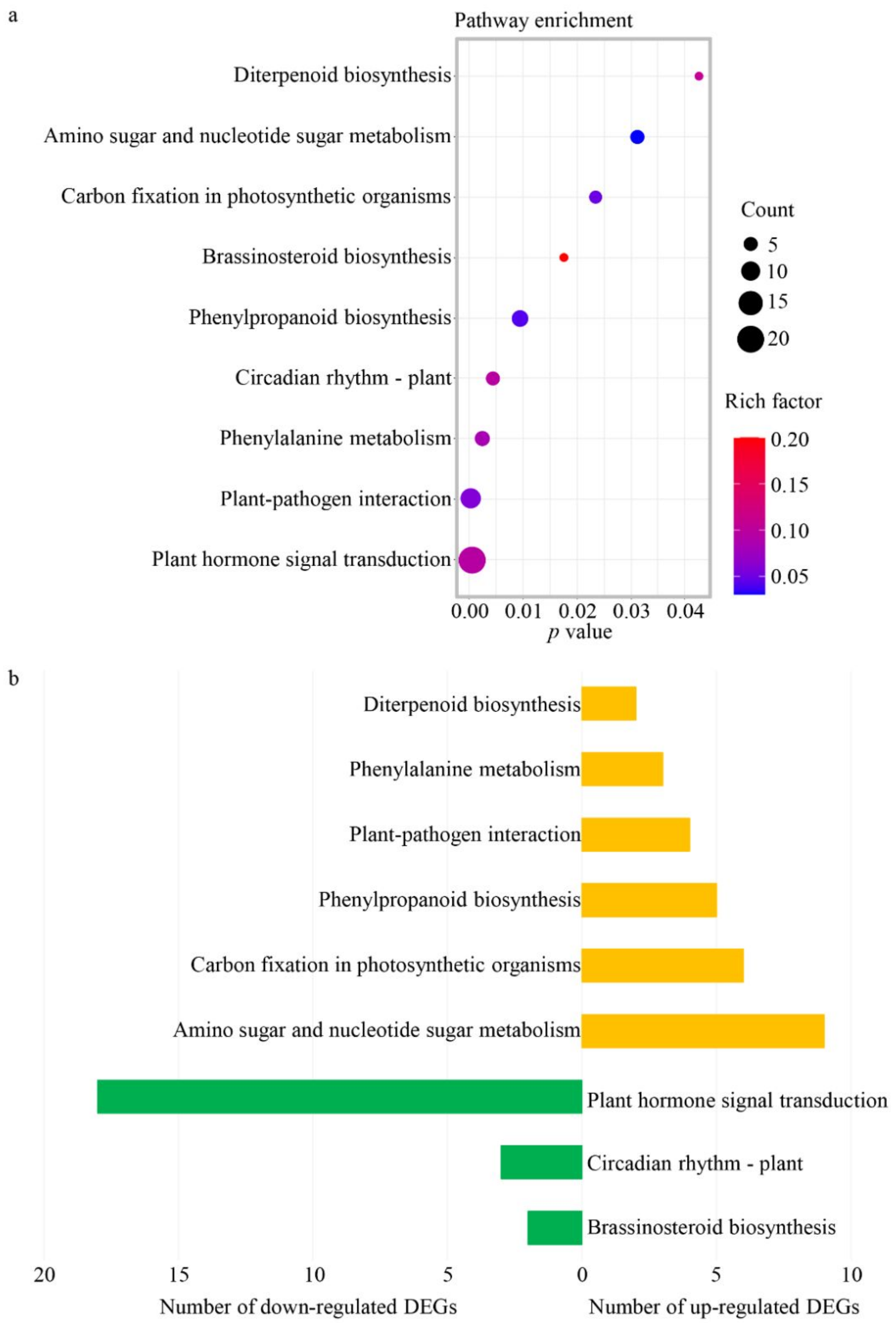

Supplementary Fig. 2 KEGG pathway enrichment of DEGs. (a) All DEGs, and (b) separated into upregulated and downregulated groups. Orange and green bars represent upregulated and downregulated DEGs, respectively. Pathway enrichment cutoff: $p < 0.05$.
