# Supplementary material for: Salt stimulates carbon fixation in the halophyte Nitraria sibirica to enhance growth
Source: For Res (Fayettev). 2025 Feb 25;5:e004. doi: 10.48130/forres-0025-0004 (PMC11922184; doi:10.48130/forres-0025-0004)
Supplement: Supplementary file 1 — Supplementary data to this article can be found online. [file forres-0025-0004-Supplementary.zip › 10.48130_forres-0025-0004-Suppl-FigureS3.pdf]

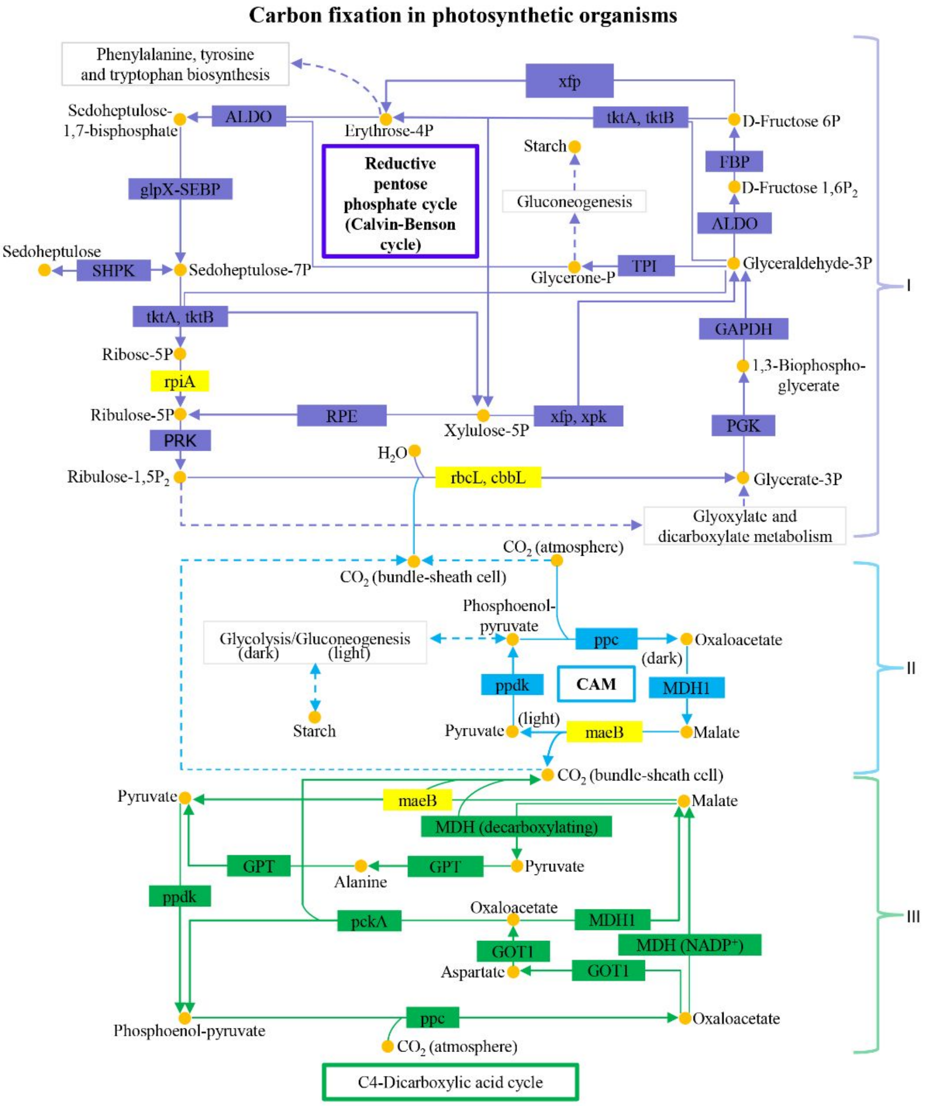

Supplementary Fig. 3 DEGs involved in carbon fixation pathways. Genes involved in the Calvin-Benson cycle (*rpiA*, *rbcL*), Crassulacean acid metabolism (CAM) and the C4-dicarboxylic acid cycle (*maeB*) were significantly upregulated under salt stress, as illustrated in network diagrams with different color codings for each pathway.
