# Supplementary material for: Salt stimulates carbon fixation in the halophyte Nitraria sibirica to enhance growth
Source: For Res (Fayettev). 2025 Feb 25;5:e004. doi: 10.48130/forres-0025-0004 (PMC11922184; doi:10.48130/forres-0025-0004)
Supplement: Supplementary file 1 — Supplementary data to this article can be found online. [file forres-0025-0004-Supplementary.zip › 10.48130_forres-0025-0004-Suppl-FigureS4.pdf]

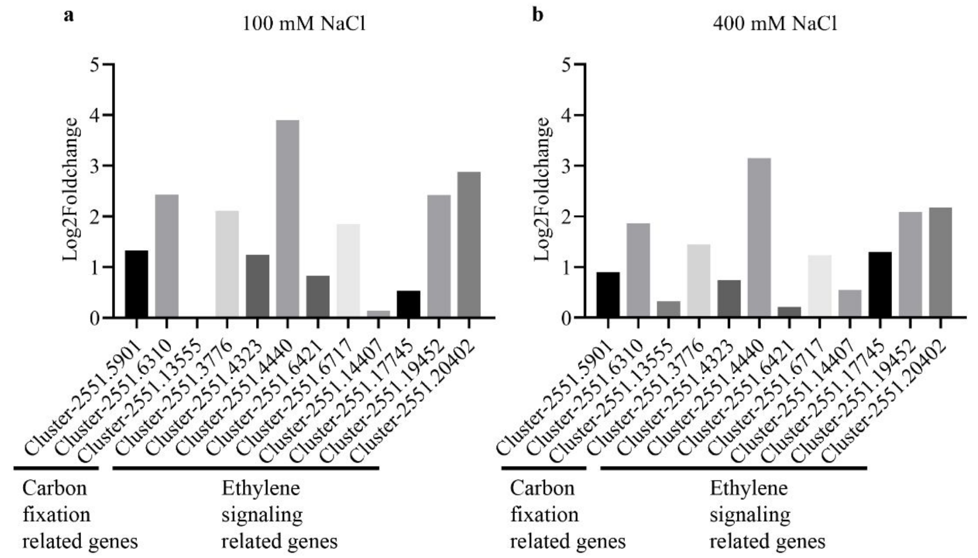

Supplementary Fig. 4. DEGs involved in photosynthesis and ethylene signaling respond positively to 100 mM NaCl and 400 mM NaCl treatment. (a) Upregulated DEGs identified from seedlings treated with 500 mM NaCl for 1 hour showed positive Log_2Fold change values under 100 mM NaCl treatment. (b) Similarly, these DEGs exhibited positive Log_2 fold change values in plants treated with 400 mM NaCl for three days.
